# Supplementary material for: Parasitized or non-parasitized, why? A study of factors influencing tick burden in roe deer neonates
Source: PLoS One. 2022 Jul 18;17(7):e0262973. doi: 10.1371/journal.pone.0262973 (PMC9292122; doi:10.1371/journal.pone.0262973)
Supplement: S1 Appendix — (PDF) [file pone.0262973.s001.pdf]

Supplementary material of: Parasitized or non-parasitized, why? A study of factors influencing tick burden in roe deer neonates.

Léa Bariod & Sonia Saïd & Clément Calenge & Stéphane Chabot &
Vincent Badeau & Gilles Bourgoin

Contents

1 Model description **3**

1.1 General model . . . 3

1.2 Submodel of $\mu(\mathbf{x}_j)$ . . . 4

1.3 Integration of $\lambda(t)$ . . . 4

1.4 The individual random effect ζ_j . . . 6

1.5 An ordinal response variable . . . 6

2 Model fit **7**

2.1 Fit with the package `nimble` . . . 7

2.2 MCMC chain mixing . . . 10

2.3 Goodness of fit . . . 11

2.4 Estimated parameters . . . 13

2.5 Explained variation in the response variable . . . 16

2.6 Expected number of ticks with age . . . 18

2.7 The years random effects . . . 19

3 Effect of other environmental variables **20**

3.1 Effect of temperature . . . 20

3.2 Effect of roe deer density . . . 22

3.3 Effect of habitat type . . . 24

Introduction

This vignette serves as the supplementary material of the paper of Bariod et al. (in prep.): *Parasitized or non-parasitized, why? A study of factors influencing tick burden in roe deer neonates*. The aim of this paper is to develop a model of the tick infestation process in roe deer fawns in the forest of Trois-Fontaines (France) between 1992 and 2018. A companion package named `tickTF` contains the data and functions used for this paper, and is required to reproduce the calculations in this document. To install this package, first install the package `devtools` and use the function `install_github` to install `tickTF`:

```
## If devtools is not yet installed, type
install.packages("devtools")

## Install the package caperpyogm
devtools::install_github("ClementCalenge/tickTF", ref="main")
```

Remark: on Windows, it is required to also install the Rtools (`https://cran.r-project.org/bin/windows/Rtools/`) on your computer to have a working `devtools` package (see `https://www.r-project.org/nosvn/pandoc/devtools.html`).

It is supposed throughout this vignette that the reader is familiar with the paper of Bariod et al., and in particular with the data collected to fit the model. Nevertheless, we present a brief reminder of the dataset used to fit the model.

The dataset is named `fticks` and is lazy-loaded when the package is loaded:

```
library(tickTF)

## Our dataset
head(fticks)

##   ticks year lothar age     bsa temperature humidity
## 1     1 1992      1   1 0.15874      11.740   74.700
## 2     1 1992      1   2 0.15340      17.140   64.560
## 3     1 1992      1   4 0.17424      19.340   57.300
## 4     1 1992      1   3 0.15340      17.400   54.120
## 5     1 1992      1   2 0.16915      17.400   54.120
## 6     1 1992      1   4 0.17424      17.041   62.926
##   habitat density
## 1       5     236
## 2       1     236
## 3       1     236
## 4       5     236
## 5       5     236
## 6       5     236
```

Every year from 1992 to 2018, the French organization for biodiversity (*Office francais de la biodiversité*) has carried out captures of roe deer fawns from April to June, and the tick burden of each animal has been assessed by the field workers (stored in the variable `ticks` of the data.frame). Three levels of tick burden are defined:

1. less than 10 ticks
2. between 10 and 20 ticks
3. more than 20 ticks

For each captured fawn, the following basic variables have been reported:

- `age`: the age of the fawn at the time of capture (in days);
- `temperature`: the mean temperature during the 6 days preceding the capture;
- `humidity`: the mean humidity during the 6 days preceding the capture;
- `year`: the year of capture;
- `bsa`: the body surface area;
- `lothar`: the period to which the year of capture belong (defined on the basis of the forest structure following the hurricane Lothar). Three periods are defined: 1 corresponds to the year prior to the Lothar hurricane in 1999; 2 corresponds to the 10 years following the Lothar hurricane; 3 corresponds to the period 2010–2018;
- `habitat`: for animals captured from 1992 to 2014, the main habitat in the forest plot where the animal was found (1 = low scrub; 2 = high scrub; 3 = clear forest; 4 = dense and young forest; 5 = dense and older forest with continuous canopy);
- `density`: the density of the roe deer in the Trois-Fontaines Forest during the year of capture;

Our aim is to develop a model of the tick infestation process using this dataset. In a first section, we present the mathematical developments underlying the model fitted to our dataset. Then, we present how we carried out the model fit with R and the package `nimble` [VAL17] in a second section. Finally, we present alternative models that were also tested in a last section.

1 Model description

1.1 General model

We first suppose that the tick infestation process of a fawn can be described by a Poisson process: each new tick parasiting a given fawn is considered as an event, and we want to model how events occur in time. Let $\lambda_j(t)$ be the instantaneous rate of the Poisson process characterizing infestation for fawn j at age t (age is here considered as a continuous variable). The number of ticks present on the fawn j at the time of capture is the sum of all ticks acquired during its lifetime. Let a_j be the age of fawn j at the time of capture. Our Poisson process model implies that the expected number of ticks parasiting fawn j at the time of capture is equal to:

$$\Lambda_j = \int_0^{a_j} \lambda_j(t)dt \tag{1}$$

Under our model, the actual number of ticks N_j on fawn j is supposed to be drawn from a Poisson distribution with parameter Λ_j:

$$N_j \sim \mathcal{P}(\Lambda_j)$$

Now, we propose the following model of the instantaneous infestation rate $\lambda(t)$:

$$\lambda_j(t) = \zeta_j \times \mu(\mathbf{x}_j) \times s_j(t) \times (\exp(\gamma_v \times I(t > 5)) \tag{2}$$

Where:

- ζ_j is a random effect characterizing animal j. These random effects account for the different sensibilities of fawns to parasitism by ticks;
- $\mu(\mathbf{x}_j)$ is the average instantaneous infestation rate *per unit of body surface area*. This rate depends on variables $\mathbf{x}_j$ characterizing the time of the capture of animal j (see next subsection);

- $s_j(t)$ is the body surface area of animal j at time t;
- γ_v is used to alter the infestation rate of fawns by ticks after 5 days; $I(t > 5)$ takes the value 1 when t is greater than 5 and 0 otherwise. Indeed, after 5 days of engorgement, ticks start to detach themselves and fall off, leading to a decrease in the number of ticks on the animal (see paper). Thus, from the 6th day, the infestation rate starts to decrease, and γ_v accounts for this decrease.

We will need to estimate each component of this model using our dataset.

1.2 Submodel of $\mu(\mathbf{x}_j)$

As noted above, we will need a submodel of the instantaneous infestation rate $\mu(\mathbf{x}_j)$ per unit of body surface area. We noted that this rate may depend on different variables characterizing the time of capture. More precisely, we suppose:

$$\log \mu(\mathbf{x}_j) = \alpha_0 + \delta_{u(j)} + \sum_{k=1}^{p} \gamma_p x_{jk} \quad (3)$$

where α_0 is an intercept, $\{\delta_{u(j)}\}$ are random effects characterizing the year $u(j)$ of capture of the animal j (see below), and the $\{\gamma_p\}$ are a set of coefficients describing the effect of environmental variables x_{jk} on the instantaneous infestation rate. Actually, the final model include only one variable (the mean humidity during the 6 days preceding the capture), but we tried to test the effect of other variables in section 3 (temperature, roe deer density, habitat type).

The coefficients $\{\delta_{u(j)}\}$ are random effects describing the effect of the year $u(j)$ of the capture of fawn j. For a given year $u(j)$, we suppose that $\delta_{u(j)}$ is a Gaussian random effect:

$$\delta_{u(j)} \sim \mathcal{N}(0, \sigma_u^{(p)})$$

Here, the standard deviation of the distribution of the year random effects depends on the period (p) defined in the variable `lothar`. Preliminary versions of this model indeed revealed that this variance was greater just after the Lothar hurricane.

In a later section, we assess the effect of other variables on the infestation rate (average humidity, temperature, etc.), by adding effects of other variables to equation 3 (see section 3).

1.3 Integration of $\lambda(t)$

To calculate Λ_j, we need to integrate $\lambda(t)$ over the time (see equation 1). We therefore need to integrate the instantaneous rate $\lambda(t)$, which is defined in equation 2. For this, we need to know how the body surface area $s_j(t)$ of every fawn varies with time t from their birth until time of capture a_j. We propose to use the following quadratic regression model for this body surface area:

$$s_j(t) = \beta_0 + \beta_1 \times t + \beta_2 \times t^2 + \epsilon_j \quad (4)$$

With the residual ϵ_j supposed to be drawn from a Gaussian distribution:

$$\epsilon_j \sim \mathcal{N}(0, \sigma_s)$$

Indeed, we can display the relationship between age and body surface area at the time of capture in our dataset:

```
plot(fticks$age, fticks$bsa, xlab="Age", ylab="Body surface area")
ag <- seq(0,8,length=100)
lines(ag, predict(loess(bsa~age, data=fticks),
                  newdata=data.frame(age=ag)), col="red", lwd=2)
```

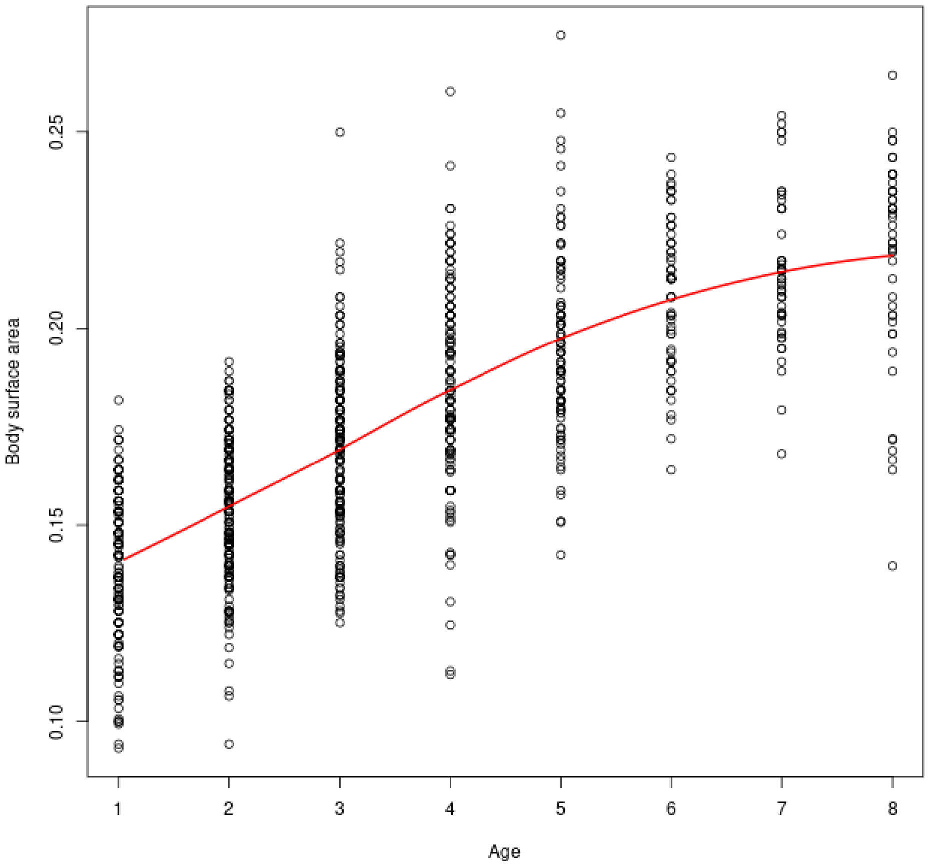

A loess smoothing suggest that the proposed quadratic model is reasonable, and that the hypothesis of homoscedasticity (variance of residuals which does not depend on age) is also satisfied.

Under this model, we can develop the equation 1 and develop the integration:

$$\begin{aligned}\Lambda_j &= \int_0^{a_j} \lambda_j(t)dt \\ &= \int_0^{a_j} \zeta_j \times \mu(\mathbf{x}_j) \times s_j(t) \times (\exp(\gamma_v \times I(t>5))dt \\ &= \zeta_j \times \mu(\mathbf{x}_j) \int_0^{a_j} s_j(t) \times \exp(\gamma_v \times I(t>5))dt\end{aligned}$$

Therefore, to calculate Λ_j, we need to calculate the expression E_j:

$$E_j = \int_0^{a_j} s_j(t) \times \exp(\gamma_v \times I(t>5))dt$$

We need to distinguish two cases:

- Either the fawn j is captured before he reaches the age of 6 days, in which case:

$$E_j = \int_0^{a_j} s_j(t)$$

- Or the captured fawn is older than 5 days and we need to account for the decrease in the infestation rate after 5 days:

$$E_j = \int_0^{5} s_j(t) + \exp(\gamma_v) \times \int_5^{a_j} s_j(t)$$

In both cases, to calculate E_j, we need to integrate $s_j(t)$ (though on different intervals). We therefore need to find the antiderivative of $s_j(t)$. The function $s_j(t)$ is given by equation 4, so that the antiderivative $S_j(t)$ of $s_j(t)$ is:

$$S_j(t) = \beta_0 \times t + \frac{\beta_1}{2} \times t^2 - \frac{\beta_2}{3} \times t^3 + \epsilon_j \times t$$

We can now estimate the expected number of ticks on fawn j:

- for fawn of 5 days or less:

$$\Lambda_j = \zeta_j \times \mu(\mathbf{x}_j) \times \left(\beta_0 \times a_j + \frac{\beta_1}{2} \times a_j^2 + \frac{\beta_2}{3} \times a_j^3 + \epsilon_j \times a_j \right)$$

- for older fawns:

$$\Lambda_j = \zeta_j \times \mu(\mathbf{x}_j) \times \Bigg\{ \left(5\beta_0 + 12.5\beta_1 + \frac{125}{3}\beta_2 + 5\epsilon_j \right) + \exp(\gamma_v) \times \Bigg(\beta_0 \times a_j + \frac{\beta_1}{2} \times a_j^2 + \frac{\beta_2}{3} \times a_j^3 + \epsilon_j \times a_j - (5\beta_0 + 12.5\beta_1 + \frac{125}{3}\beta^2 + 5\epsilon_j) \Bigg) \Bigg\} \quad (5)$$

1.4 The individual random effect ζ_j

To summarize, the number of ticks found on the fawn j is the result of progressive accumulation of ticks from birth until capture. The average instantaneous infestation rate is the product of an instantaneous infestation rate – which, (i) depends on whether the animal is older than 5 days or not, and (ii) accounts for the body surface area of the fawn – and of a random individual effect ζ_j, which characterizes each animal j (see equation 2).

Now, let us consider this random effect ζ_j. We suppose that this random effect is drawn from a Gamma distribution with parameters:

$$\zeta_j \sim \mathcal{G}(1/\phi, \phi)$$

Here, the parameter ϕ controls the variance of individual random effects (the variance of these random effects will be large if ϕ is small). It is well known that if the number of ticks N_j on a fawn j follows a Poisson distribution with a parameter Λ_j randomly drawn from a gamma distribution, then the distribution of N_j is negative binomial with parameters:

$$N_j \sim \mathcal{NB}(\Omega_j, \phi)$$

with $\zeta_j \times \Omega_j = \Lambda_j$ (see [VEN02]).

1.5 An ordinal response variable

The model developed in the previous subsections uses the number of ticks N_j detected on a captured fawn as a response variable. Actually, we do not know the exact value of N_j, as the response variable was a class of abundance of ticks was reported for each one (see introduction). Let Y_j be a variable defined by:

- $Y_j = 1$ when $N_j < 10$
- $Y_j = 2$ when $10 \leq N_j \leq 20$
- $Y_j = 3$ when $N_j > 20$

In our data, this variable corresponds to the column `ticks` of the data.frame `fticks`. This is our actuar response variable.

Now, let $F(N; \Omega_j, \phi)$ be the cumulative distribution function of a variable N following the negative binomial distribution with parameters Ω_j and ϕ. Then:

$$
\begin{aligned}
P(Y_j = 1) &= F(9; , \Omega_j) \\
P(Y_j = 2) &= F(20, \Omega_j) - F(9, \Omega_j) \\
P(Y_j = 3) &= 1 - F(20, \Omega_j)
\end{aligned}
$$

We programmed this distribution for the package `nimble` to allow the model fit. We describe this fit in the following section.

2 Model fit

2.1 Fit with the package `nimble`

We fitted this model with the package `nimble` for the R software. We used MCMC to fit this model using the following prior distributions for the parameters:

$$
\begin{aligned}
\alpha_0 &\sim \mathcal{N}(0, 20) \\
\beta_0 &\sim \mathcal{N}(0, 20) \\
\beta_1 &\sim \mathcal{U}(0, 1) \\
\beta_2 &\sim \mathcal{N}(0, 20) \\
\gamma_v &\sim \mathcal{N}(0, 20) \\
\sigma_u^{(p)} &\sim \mathcal{U}(0, 100) \\
\sigma_s &\sim \mathcal{U}(0, 100) \\
\phi &\sim \mathcal{U}(0, 100)
\end{aligned}
$$

As noted in the previous section, the probability distribution for the variable Y_j is not available in the package `nimble`. We therefore programmed it using the functions of this package (see [VAL21] for a description of how to add a distribution to the package `nimble`). The code used to add the distribution of Y_j for MCMC sampling is displayed below:

```
library(nimble)

## Function giving the probability of Y_j (here passed as argument x)
## given the value of Omega (here passed as argument lambda) and phi

dticks <- nimbleFunction(
    run=function(x=integer(0), lambda=double(0, default=1),
                 phi=double(0, default=1),
                 log = integer(0, default = 0)) {
    returnType(double(0))
    proba <- phi/(phi+lambda)
    p1 <- pnbinom(9, size=phi, prob=proba,
                  lower.tail = TRUE, log.p = TRUE)
    p3 <- pnbinom(20, size=phi, prob=proba,
                  lower.tail = FALSE, log.p = TRUE)
    p2 <- log(0.0000001+
              pnbinom(20, size=phi, prob=proba,
                      lower.tail = TRUE, log.p = FALSE)-
              pnbinom(9, size=phi, prob=proba,
                      lower.tail = TRUE, log.p = FALSE))
    lpro <- (c(p1,p2,p3)[x])
    if (log) return(lpro)
```

```
    else return(exp(lpro))
})


## Function needed to simulate random values from the probability distribution
## of Y_j, as a function of Omega (here passed as lambda) and phi.
rticks <- nimbleFunction(
    run = function(n = integer(0), lambda=double(0, default=1),
                   phi=double(0, default=1)) {
        returnType(double(0))
        if(n != 1) print("rtiques only allows n = 1; using n = 1.")
        u <- rnbinom(1, size=phi, prob=phi/(phi+lambda))
        if (u<10) {
            return(1)
        }
        if(u<21) {
            return(2)
        }
        return(3)
    })
```

We then programmed the model described in the following sections for `nimble`, and prepared the data for the fit:

```
codePaperModel <- nimbleCode({

    ## Priors
    intercept ~ dnorm(0,sd=20)
    gammah ~ dnorm(0,sd=20)
    gammav ~ dnorm(0,sd=20)
    beta0p ~ dnorm(0,sd=20)
    beta1p ~ dnorm(0,sd=20)
    beta2p ~ dnorm(0,sd=20)
    for (i in 1:3) {
        sigma_u[i]~dunif(0,100)
    }
    sigma_s~dunif(0,100)
    phi~dunif(0,100);


    ## Year random effects
    for (i in 1:nyears) {
        delta_u[i] ~ dnorm(intercept, sd=sigma_u[pery[i]]);
    }

    ## Because we work on centred data for body surface area
    ## (for better mixing)
    beta0 <- beta0p - beta1p*xbar + beta2p*pow(xbar,2.0)
    beta1 <- beta1p - 2*beta2p*xbar
    beta2 <- beta2p


    ## Integration of body surface area
    for (i in 1:nobs) {

        ## Expected bsa
        yhat[i] <- beta0p + beta1p*AGEc[i] + beta2p*pow(AGEc[i],2.0)
```

```r
        SURFACE[i] ~ dnorm(yhat[i], sd=sigma_s)
        epsilon[i] <- SURFACE[i] - yhat[i]

        ## Integration
        Ej[i] <- step(5-AGE[i])*(beta0*AGE[i] + (beta1/2.0)*pow(AGE[i],2.0) +
                                 (beta2/3.0)*pow(AGE[i],3.0) +
                                 epsilon[i]*AGE[i]) +
            (1-step(5-AGE[i]))*((5.0*beta0 + 12.5*beta1 + (125.0/3.0)*beta2 + 5.0*epsilon[i]) +
                                exp(gammav)*(beta0*AGE[i] + (beta1/2.0)*pow(AGE[i],2.0) +
                                             (beta2/3.0)*pow(AGE[i],3.0) +
                                             epsilon[i]*AGE[i]) -
                                exp(gammav)*(5.0*beta0 + 12.5*beta1 +
                                             (125.0/3.0)*beta2 + 5.0*epsilon[i]))

        ## Expectation
        Lambdaj[i] <- Ej[i] * exp(delta_u[YEAR[i]]+gammah*HUMIDITY[i])

        ## Likelihood
        TICK_CLASS[i]~dticks(Lambdaj[i], phi)
    }

})


## Year as an integer variable
int_year <- as.integer(fticks$year-(min(fticks$year)-1))

## period for each year
yl <- unique(fticks[,c("year","lothar")])
pery <- yl$lothar[order(yl$year)]


## Constants
TicksConst <- list(nobs=nrow(fticks), nyears=max(int_year), YEAR=int_year, pery=pery)

## Data
TicksData <- list(TICK_CLASS=as.integer(fticks$ticks),
                  AGE=fticks$age,
                  xbar=mean(fticks$age),
                  AGEc=fticks$age-mean(fticks$age),
                  SURFACE=fticks$bsa,
                  HUMIDITY=fticks$humidity-mean(fticks$humidity))

## Starting values
inits <- list(beta0p = 0.17, beta1p = 0.01, beta2p = -0.0007,
              gammav = -0.8, gammah=0, intercept = 2.8, phi = 0.79,
              sigma_u = c(0.64, 0.64, 0.64), sigma_s = 0.02,
              delta_u = c(2.07, 2.91, 2.79,
                          2.82, 2.57, 3.47, 2.85, 2.77, 2.59, 2.4, 1.45, 3.01, 2.5,
                          2.68, 2.76, 2.76, 2.71, 3.41, 2.43, 2.4, 2.59, 2.19, 2.63,
                          1.72, 2.12, 2.59, 2.19))

## We use the same for the 4 chains
linits <- list(inits,inits,inits,inits)
```

Finally, we used the function `nimbleMCMC` to fit the model. We used 4 chains of 50000 iterations after a burn-in period of 1000 samples. To save some memory space, we thinned the chains by selecting one

sample every 20 iterations. WARNING: THIS CALCULATION TAKES A VERY LONG TIME (more than one hour) !!!! Note that we have included the results of this calculation as a dataset of the package, so that the reader does not need to launch this function to reproduce the other calculations:

```
set.seed(123)
resultsModel <-
    nimbleMCMC(code = codePaperModel, constants = TicksConst,
               data = TicksData, inits = linits,
               nchains = 4, niter = 51000, nburnin=1000, thin=20,
               summary = TRUE, samplesAsCodaMCMC=TRUE,
               monitors = c("delta_u","gammav", "gammah", "sigma_u",
                            "intercept",
                            "phi","sigma_s","beta0p","beta1p","beta2p"))
```

2.2 MCMC chain mixing

Once the MCMC samples have been obtained, we can plot the chain for a visual examination of the mixing for the top parameters:

```
library(bayesplot)
mcmc_trace(resultsModel$samples,
           regex_pars=c("intercept", "phi", "sigma_s","sigma_u","gammav","gammah",
                        "beta0p","beta1p","beta2p"))
```

We also calculate the diagnostic of Gelman & Rubin (see [GEL92]) to assess the mixing properties:

```
library(coda)
gelman.diag(resultsModel$samples)

## Potential scale reduction factors:
##
##               Point est. Upper C.I.
## beta0p                 1       1.01
## beta1p                 1       1.00
## beta2p                 1       1.01
## delta_u[1]             1       1.00
## delta_u[2]             1       1.00
## delta_u[3]             1       1.00
## delta_u[4]             1       1.00
## delta_u[5]             1       1.00
## delta_u[6]             1       1.00
## delta_u[7]             1       1.00
## delta_u[8]             1       1.00
## delta_u[9]             1       1.00
## delta_u[10]            1       1.00
## delta_u[11]            1       1.00
## delta_u[12]            1       1.00
## delta_u[13]            1       1.00
## delta_u[14]            1       1.00
## delta_u[15]            1       1.00
## delta_u[16]            1       1.00
## delta_u[17]            1       1.00
## delta_u[18]            1       1.00
## delta_u[19]            1       1.00
## delta_u[20]            1       1.00
```

```
## delta_u[21]          1       1.01
## delta_u[22]          1       1.00
## delta_u[23]          1       1.00
## delta_u[24]          1       1.00
## delta_u[25]          1       1.00
## delta_u[26]          1       1.00
## delta_u[27]          1       1.00
## gammah               1       1.00
## gammav               1       1.00
## intercept            1       1.00
## phi                  1       1.00
## sigma_s              1       1.00
## sigma_u[1]           1       1.01
## sigma_u[2]           1       1.00
## sigma_u[3]           1       1.01
##
## Multivariate psrf
##
## 1.01
```

Mixing properties are excellent here (the visual examination of the chains show a good mixing, and the diagnostic of Gelman and Rubin is never above 1.02).

2.3 Goodness of fit

We then check the goodness of fit of the model. As for the count model, we simulate M virtual datasets (one per MCMC sample) and consider several summary statistics (see below). For each statistic, we compare the observed value (calculated on the actual dataset) with the distribution of values calculated on the simulated datasets. We first use the function `simulatePaperModel` to simulate the datasets using the MCMC samples (see the help page of this function):

```
simulatedData <- simulatePaperModel(resultsModel$samples, TicksData, TicksConst)
```

We first calculated the proportion of fawns belonging to the three classes of ticks ($Y_j = 1, 2$ or 3) in the observed dataset as well as in each simulated dataset. We used the distribution of simulated proportion of fawns in the three classes to derive a 95% credible interval on these proportions:

```
## Observed:
sapply(1:3,function(i) mean(TicksData$TICK_CLASS==i))

## [1] 0.7212435 0.1730570 0.1056995

## Simulated credible intervals
sapply(1:3, function(i) {quantile(rowMeans(simulatedData==i),c(0.025, 0.975))})

##             [,1]      [,2]       [,3]
## 2.5%  0.6922280 0.1233161 0.09740933
## 97.5% 0.7637306 0.1782383 0.14715026
```

The observed values are within the limits of the credible intervals for all classes.

We also calculated, for each possible age from 1 day old to 8 days old, the proportion of fawns in each class of ticks in our dataset, as well as the 95% credible interval on this proportion derived from our simulated datasets:

```r
## Class 1: are all the observed proportion within the limits of the 95% CI?
observed <- (tapply(TicksData$TICK_CLASS==1, TicksData$AGE, mean))
sim <- apply(simulatedData,1,function(x) tapply(x==1, TicksData$AGE, mean))
qs <- apply(sim,1,function(x) quantile(x, c(0.025,0.975)))
(observed>=qs[1,]&observed<=qs[2,])

##    1    2    3    4    5    6    7    8
## TRUE TRUE TRUE TRUE TRUE TRUE TRUE TRUE

## Class 2: are all the observed proportion within the limits of the 95% CI?
observed <- (tapply(TicksData$TICK_CLASS==2, TicksData$AGE, mean))
sim <- apply(simulatedData,1,function(x) tapply(x==2, TicksData$AGE, mean))
qs <- apply(sim,1,function(x) quantile(x, c(0.025,0.975)))
(observed>=qs[1,]&observed<=qs[2,])

##     1     2     3     4     5     6     7     8
##  TRUE  TRUE  TRUE  TRUE  TRUE  TRUE  TRUE FALSE

## Class 3: are all the observed proportion within the limits of the 95% CI?
observed <- (tapply(TicksData$TICK_CLASS==3, TicksData$AGE, mean))
sim <- apply(simulatedData,1,function(x) tapply(x==3, TicksData$AGE, mean))
qs <- apply(sim,1,function(x) quantile(x, c(0.025,0.975)))
(observed>=qs[1,]&observed<=qs[2,])

##    1    2    3    4    5    6    7    8
## TRUE TRUE TRUE TRUE TRUE TRUE TRUE TRUE
```

The observed proportion is, in all cases but one, within the limits of the 95% credible interval.

We also calculated the proportion of animals in each class for each year of our dataset, and we derived a 95% credible interval for each proportion from the distribution of our simulated datasets. For each class, we calculated the proportion of credible intervals including the observed value:

```r
## Class 1
ry <- apply(simulatedData, 1, function(x) tapply(x, TicksConst$YEAR, function(y) mean(y==1)))
rg <- apply(ry,1,quantile,c(0.025,0.975))
ta <- tapply(TicksData$TICK_CLASS, TicksConst$YEAR, function(y) mean(y==1))
mean(ta>=rg[1,]&ta<=rg[2,])

## [1] 1

## Class 2
ry <- apply(simulatedData, 1, function(x) tapply(x, TicksConst$YEAR, function(y) mean(y==2)))
rg <- apply(ry,1,quantile,c(0.025,0.975))
ta <- tapply(TicksData$TICK_CLASS, TicksConst$YEAR, function(y) mean(y==2))
mean(ta>=rg[1,]&ta<=rg[2,])

## [1] 0.9259259

## Class 3
ry <- apply(simulatedData, 1, function(x) tapply(x, TicksConst$YEAR, function(y) mean(y==3)))
rg <- apply(ry,1,quantile,c(0.025,0.975))
ta <- tapply(TicksData$TICK_CLASS, TicksConst$YEAR, function(y) mean(y==3))
mean(ta>=rg[1,]&ta<=rg[2,])
```

```
## [1] 1
```

Therefore, all the tests indicate a good fit of our model.

2.4 Estimated parameters

We present the estimated coefficients of the model below:

```
ro <- round(resultsModel$summary$all.chains,3)
ro[-grep("delta",rownames(ro)),]

##                Mean  Median St.Dev. 95%CI_low 95%CI_upp
## beta0p        0.176   0.176   0.001     0.174     0.178
## beta1p        0.014   0.014   0.000     0.013     0.014
## beta2p       -0.001  -0.001   0.000    -0.001    -0.001
## gammah        0.010   0.010   0.008    -0.006     0.025
## gammav      -17.647 -15.068  11.850   -46.606    -2.598
## intercept     2.853   2.855   0.085     2.675     3.019
## phi           0.676   0.672   0.082     0.529     0.846
## sigma_s       0.021   0.021   0.000     0.020     0.022
## sigma_u[1]    0.438   0.392   0.281     0.044     1.141
## sigma_u[2]    0.822   0.769   0.297     0.422     1.543
## sigma_u[3]    0.165   0.140   0.124     0.011     0.462
```

For clarity, the 27 year random effects have been removed from this table. We note that the effect of humidity (`gammah`) is not far from being significantly different from 0 at the level 5%. We tried to fit this model again after removal of the effect of humidity, to allow a comparison of the WAIC of the two models. We first programmed this model:

```
codePaperModel0 <- nimbleCode({

    ## Priors
    intercept ~ dnorm(0,sd=20)
    gammav ~ dnorm(0,sd=20)
    beta0p ~ dnorm(0,sd=20)
    beta1p ~ dnorm(0,sd=20)
    beta2p ~ dnorm(0,sd=20)
    for (i in 1:3) {
        sigma_u[i]~dunif(0,100)
    }
    sigma_s~dunif(0,100)
    phi~dunif(0,100);


    ## Year random effects
    for (i in 1:nyears) {
        delta_u[i] ~ dnorm(intercept, sd=sigma_u[pery[i]]);
    }

    ## Because we work on centred data for body surface area
    ## (for better mixing)
    beta0 <- beta0p - beta1p*xbar + beta2p*pow(xbar,2.0)
    beta1 <- beta1p - 2*beta2p*xbar
    beta2 <- beta2p
```

```
    ## Integration of body surface area
    for (i in 1:nobs) {

        ## Expected bsa
        yhat[i] <- beta0p + beta1p*AGEc[i] + beta2p*pow(AGEc[i],2.0)
        SURFACE[i] ~ dnorm(yhat[i], sd=sigma_s)
        epsilon[i] <- SURFACE[i] - yhat[i]

        ## Integration
        Ej[i] <- step(5-AGE[i])*(beta0*AGE[i] + (beta1/2.0)*pow(AGE[i],2.0) +
                                  (beta2/3.0)*pow(AGE[i],3.0) +
                                  epsilon[i]*AGE[i]) +
            (1-step(5-AGE[i]))*((5.0*beta0 + 12.5*beta1 + (125.0/3.0)*beta2 + 5.0*epsilon[i]) +
                                exp(gammav)*(beta0*AGE[i] + (beta1/2.0)*pow(AGE[i],2.0) +
                                             (beta2/3.0)*pow(AGE[i],3.0) +
                                             epsilon[i]*AGE[i]) -
                                exp(gammav)*(5.0*beta0 + 12.5*beta1 +
                                             (125.0/3.0)*beta2 + 5.0*epsilon[i]))

        ## Expectation
        Lambdaj[i] <- Ej[i] * exp(delta_u[YEAR[i]])

        ## Likelihood
        TICK_CLASS[i]~dticks(Lambdaj[i], phi)
    }

})


TicksData0 <- TicksData
TicksData0$HUMIDITY <- NULL

## Starting values
inits <- list(beta0p = 0.17, beta1p = 0.01, beta2p = -0.0007,
              gammav = -0.8, intercept = 2.8, phi = 0.79,
              sigma_u = c(0.64, 0.64, 0.64), sigma_s = 0.02,
              delta_u = c(2.07, 2.91, 2.79,
                          2.82, 2.57, 3.47, 2.85, 2.77, 2.59, 2.4, 1.45, 3.01, 2.5,
                          2.68, 2.76, 2.76, 2.71, 3.41, 2.43, 2.4, 2.59, 2.19, 2.63,
                          1.72, 2.12, 2.59, 2.19))

## We use the same for the 4 chains
linits <- list(inits,inits,inits,inits)
```

And we then fitted the model with `nimble`. WARNING: THIS CALCULATION TAKES A VERY LONG TIME (more than one hour) !!!! Note that, as for the previous model, we also have included the results of this calculation as the dataset `resultsModel0` of the package, so that the reader does not need to launch this function to reproduce later calculations:

```
set.seed(123)
resultsModel0 <-
    nimbleMCMC(code = codePaperModel0, constants = TicksConst,
               data = TicksData0, inits = linits,
               nchains = 4, niter = 51000, nburnin=1000, thin=20,
               summary = TRUE, samplesAsCodaMCMC=TRUE,
               monitors = c("delta_u","gammav",  "sigma_u",
```

```
                              "intercept",
                              "phi","sigma_s","beta0p","beta1p","beta2p"))
```

Then, we calculated the WAIC criterion for the two models (with and without humidity), using the function `waicTicks` of the package (see the help page of this function – Warning, this calculation can last > 10 seconds):

```
withHumidity <- waicTicks(resultsModel$samples, TicksData, TicksConst)
withoutHumidity <- waicTicks(resultsModel0$samples, TicksData0, TicksConst)
```

We compare the two WAIC:

```
withHumidity$waic

##         WAIC           SE
## 1374.659224     1.574346

withoutHumidity$waic

##         WAIC           SE
## 1373.883494     1.574182
```

They are sensibly identical: both models predict the data with the same efficiency: a positive effect of the humidity is likely but cannot be ascertained (see discussion in the paper). Note moreover that all other model parameters are sensibly identical:

```
plot(resultsModel$summary$all.chains[-31,2],
     resultsModel0$summary$all.chains[,2],
     xlim=c(0,5), ylim=c(0,5), asp=1,
     xlab="Parameters of the model with humidity",
     ylab="Parameters of the model without humidity")
abline(0,1)
```

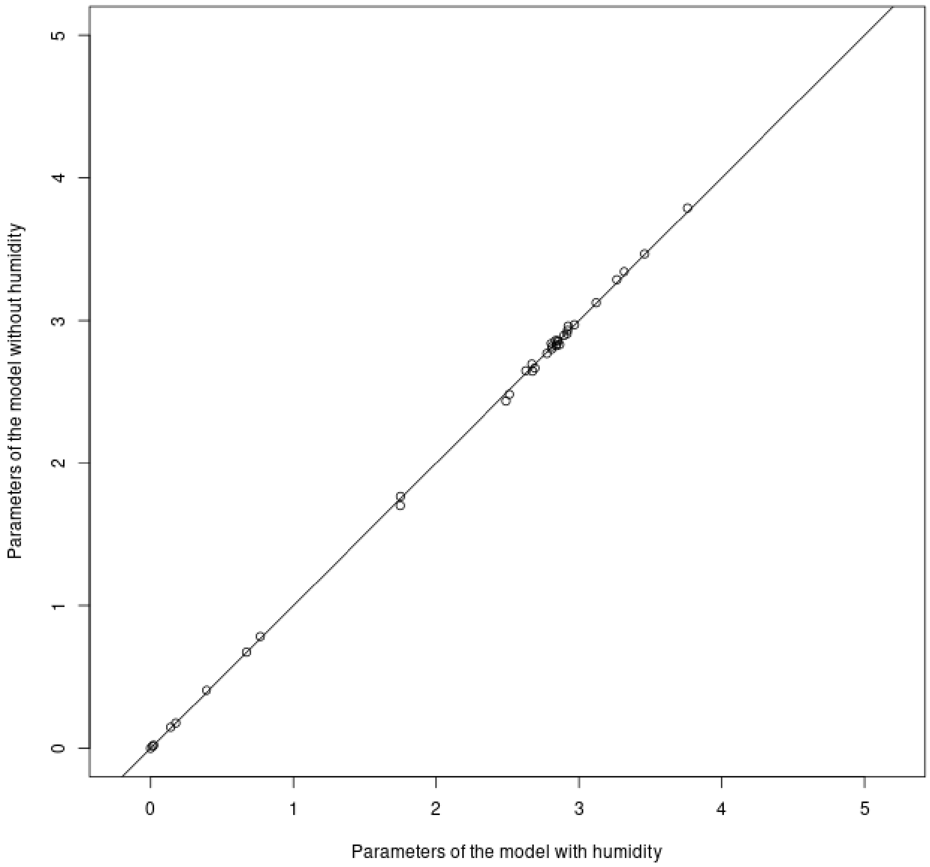

In other words, the inclusion of the effect of humidity on the instantaneous infestation rate does not affect other parameters.

2.5 Explained variation in the response variable

We used the datasets simulated in the section 2.3 to calculate the distribution of Spearman rank correlation between predicted tick burden class (taken as an ordinal variable with values from 1 to 3) and the observed tick burden class:

```
hist(apply(simulatedData,1,function(x) cor(x, TicksData$TICK_CLASS)),
     main="Spearman Correlation",
     xlab="Correlation coefficient")
```

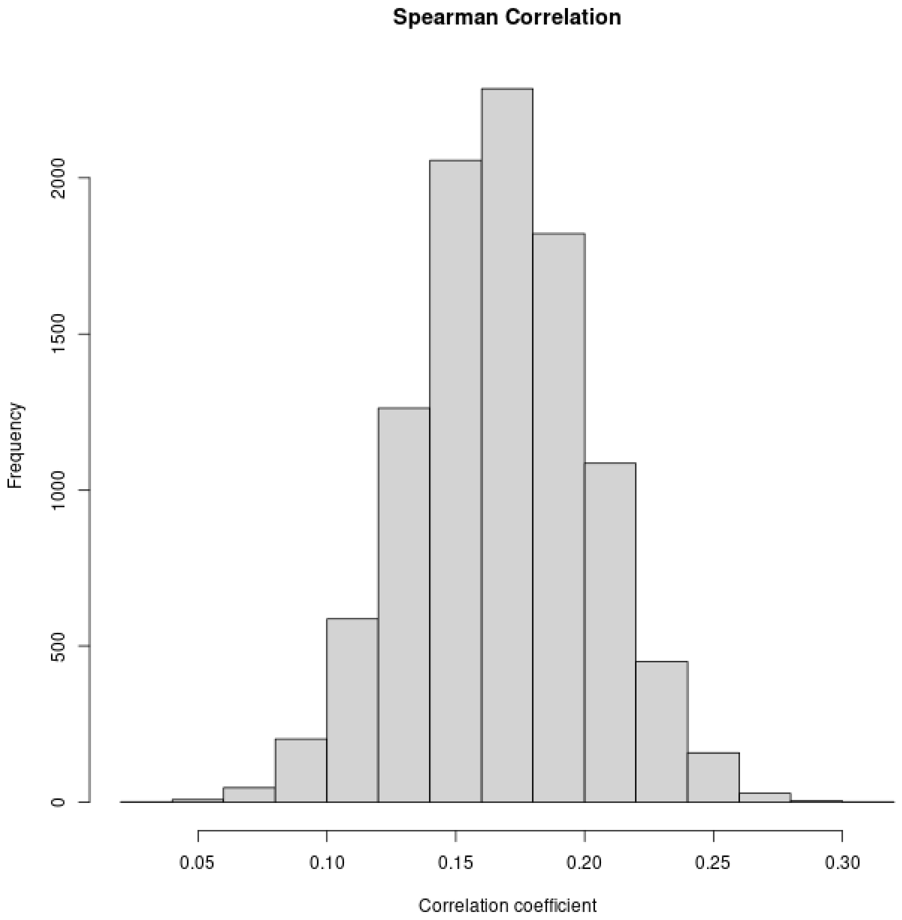

The correlation coefficients are ≈ 0.17. There is a lot of unexplained variation left in our dataset. Another interesting criterion to assess the proportion of explained variation, is the proportion of observed fawns predicted by the model to be in the correct tick burden class. For each observed class of fawns, we calculate the mean proportion of fawns for which the class was correctly predicted by the model in our simulated dataset (SE in parenthesis), and we compare this proportion to the mean proportion of fawns that would be obtained by randomly allocating the classes to fawns – keeping the frequency of the fawns constant in the datasets (SE of this distribution under random hypothesis in parentheses):

```
rand <- t(sapply(1:999, function(i) {
    x <- sample(TicksData$TICK_CLASS)
    c(mean(x[TicksData$TICK_CLASS==1]==1),
      mean(x[TicksData$TICK_CLASS>2]>2),
      mean(x[TicksData$TICK_CLASS==3]==3))
}))

obe <- t(apply(simulatedData,1,function(x) {
    c(mean(x[TicksData$TICK_CLASS==1]==1),
      mean(x[TicksData$TICK_CLASS>2]>2),
      mean(x[TicksData$TICK_CLASS==3]==3))
}))

sran <- apply(round(rand,2),2,
              function(x)
    paste0(round(mean(x),2), " (SE = ",
           round(sd(x),2), ")"))

sobe <- apply(round(obe,2),2,
```

```r
              function(x)
    paste0(round(mean(x),2), " (SE = ",
           round(sd(x),2), ")"))
data.frame(Class=1:3,
           observed=sobe,
           UnderRandomAssumption=sran)
```

```
##   Class          observed UnderRandomAssumption
## 1     1 0.77 (SE = 0.02)      0.72 (SE = 0.01)
## 2     2 0.23 (SE = 0.04)      0.11 (SE = 0.03)
## 3     3 0.23 (SE = 0.04)      0.11 (SE = 0.03)
```

This confims the results obtained with the Spearman correlation coefficient: our model is slightly better a random allocation, but a lot of unexplained variation remains in our data. This is also confirmed by the value of the parameter `phi` (see previously: $\phi \approx 0.7$): indeed, the variance of a negative binomial distribution is $\mu + \mu^2/\phi$ – where μ is the mean of the distribution. When ϕ is very large, the binomial distribution converges toward a Poisson distribution. Here, $\phi < 1$, indicating that the between-individual variance is very large: there is a lot of noise in the data.

2.6 Expected number of ticks with age

We now use the function `predictEN` from the package to calculate, for each MCMC iteration, the expected number of ticks on the "average-sized" fawn of a given age, at average humidity, during an average year:

```r
en <- predictEN(resultsModel$samples, TicksData, TicksConst)
```

We can plot the results:

```r
lam <- en$EN
lr <- range(unlist(lam))
age <- en$age
plot(age, lam[1,], ty="n", ylim=lr, xlab="Age (days)", ylab="Mean number of ticks")
tmp <- lapply(1:nrow(lam), function(i) lines(age, lam[i,], col=rgb(0.1,0.1,0.1, 0.02)))
mo <- apply(lam,2,mean)
lines(age, mo, lwd=5)
lines(age, mo, lwd=3, col="yellow")
```

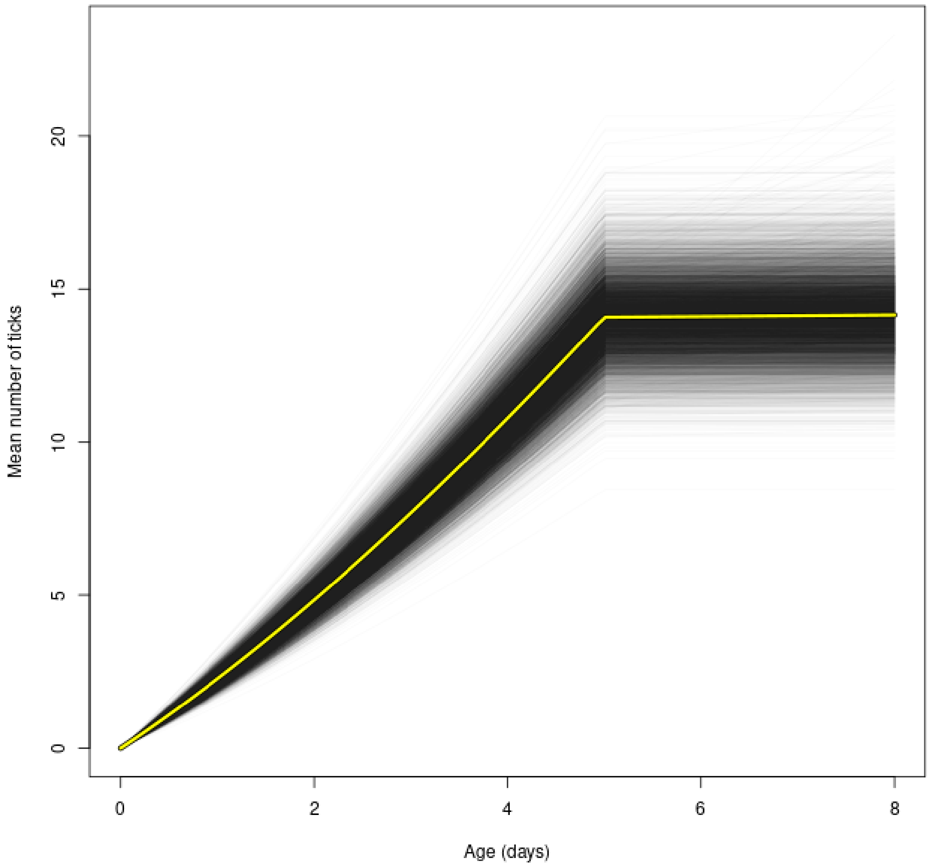

2.7 The years random effects

Finally, we present below the posterior distribution for each year random effect, using boxplots superposed to violin plots. The red lines separate the three periods. The difference of variance of random effects between the periods appears clearly on this plot:

```
library(ggplot2)
sam <- do.call(rbind,resultsModel$samples)
delt <- apply(sam[,grep("delta",colnames(sam))],2,function(x) x-sam[,"intercept"])
dfdel <- data.frame(x=factor(rep(1992:(1992+26), each=nrow(delt))), y=as.vector(delt))

plo <- ggplot(dfdel, ggplot2::aes(y = y,
                                  x = x)) +
    geom_violin(draw_quantiles = c(0.1,0.9),trim=TRUE) +
    geom_boxplot(width = 0.2, fill = "grey",
                 outlier.shape = NA)+ggplot2::ylim(c(-3,3))+
    ylab("Random effects") +
    xlab("Year")+
    geom_vline(xintercept=c(8.5,18.5),col="red", lwd=1.5)+theme_bw()

suppressWarnings(print(plo))
```

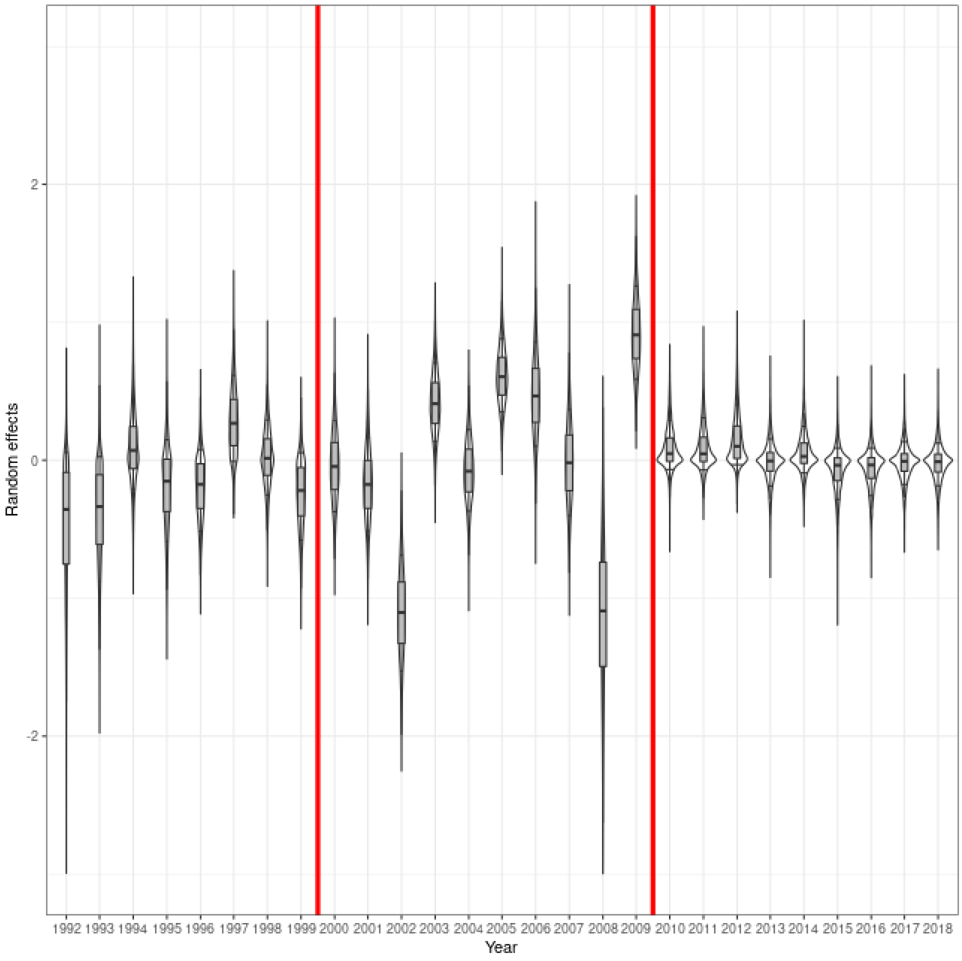

3 Effect of other environmental variables

We also tested the effect of other environmental variables, none of which had a significant effect. We give in this section the R code used for these tests.

3.1 Effect of temperature

We first test the effect of the mean temperature on the instantaneous infestation rate, by adding a term in the equation 3. We code the model allowing an additional variable in the model below:.

```
codePaperModelP1 <- nimbleCode({

    ## Priors
    intercept ~ dnorm(0,sd=20)
    gammah ~ dnorm(0,sd=20)
    gammav ~ dnorm(0,sd=20)
    gamman ~ dnorm(0,sd=20)
    beta0p ~ dnorm(0,sd=20)
    beta1p ~ dnorm(0,sd=20)
    beta2p ~ dnorm(0,sd=20)
    for (i in 1:3) {
        sigma_u[i]~dunif(0,100)
    }
    sigma_s~dunif(0,100)
```

```
    phi~dunif(0,100);


    ## Year random effects
    for (i in 1:nyears) {
        delta_u[i] ~ dnorm(intercept, sd=sigma_u[pery[i]]);
    }

    ## Because we work on centred data for body surface area
    ## (for better mixing)
    beta0 <- beta0p - beta1p*xbar + beta2p*pow(xbar,2.0)
    beta1 <- beta1p - 2*beta2p*xbar
    beta2 <- beta2p


    ## Integration of body surface area
    for (i in 1:nobs) {

        ## Expected bsa
        yhat[i] <- beta0p + beta1p*AGEc[i] + beta2p*pow(AGEc[i],2.0)
        SURFACE[i] ~ dnorm(yhat[i], sd=sigma_s)
        epsilon[i] <- SURFACE[i] - yhat[i]

        ## Integration
        Ej[i] <- step(5-AGE[i])*(beta0*AGE[i] + (beta1/2.0)*pow(AGE[i],2.0) +
                                 (beta2/3.0)*pow(AGE[i],3.0) +
                                 epsilon[i]*AGE[i]) +
            (1-step(5-AGE[i]))*((5.0*beta0 + 12.5*beta1 + (125.0/3.0)*beta2 + 5.0*epsilon[i]) +
                                exp(gammav)*(beta0*AGE[i] + (beta1/2.0)*pow(AGE[i],2.0) +
                                             (beta2/3.0)*pow(AGE[i],3.0) +
                                             epsilon[i]*AGE[i]) -
                                exp(gammav)*(5.0*beta0 + 12.5*beta1 +
                                             (125.0/3.0)*beta2 + 5.0*epsilon[i]))

        ## Expectation
        Lambdaj[i] <- Ej[i] * exp(delta_u[YEAR[i]]+gammah*HUMIDITY[i]+gamman*VARIABLES[i])


        ## Likelihood
        TICK_CLASS[i]~dticks(Lambdaj[i], phi)
    }

})


## Data
TicksDataP1 <- TicksData
TicksDataP1$VARIABLES <- fticks$temperature-mean(fticks$temperature)

## Starting values
inits$gammah <- 0
inits$gamman <- 0

## We use the same for the 4 chains
linits <- list(inits,inits,inits,inits)
```

We then fit the model with `nimble`. WARNING: THIS CALCULATION TAKES A VERY LONG TIME

(more than one hour) !!!! Note that, as for the previous model, we also have included the results of this calculation as the dataset `resultsModelTemperature` of the package, so that the reader does not need to launch this function to reproduce later calculations:

```
set.seed(123)
resultsModelTemperature <-
    nimbleMCMC(code = codePaperModelP1, constants = TicksConst,
               data = TicksDataP1, inits = linits,
               nchains = 4, niter = 51000, nburnin=1000, thin=20,
               summary = TRUE, samplesAsCodaMCMC=TRUE,
               monitors = c("delta_u","gammav", "sigma_u","gamman","gammah",
                            "intercept","phi","sigma_s","beta0p",
                            "beta1p","beta2p"))
```

The estimated parameter for the temperature is displayed below:

```
round(resultsModelTemperature$summary$all.chains["gamman",],3)

##      Mean    Median   St.Dev. 95%CI_low 95%CI_upp
##     0.000     0.000     0.021    -0.041     0.040
```

Our data do not allow to demonstrate an effect of the temperature on the infestation rate.

3.2 Effect of roe deer density

We also tested the effect of the roe deer density on the infestation rate. Indeed, this density varied a lot during the study period, as demonstrated on the plot below:

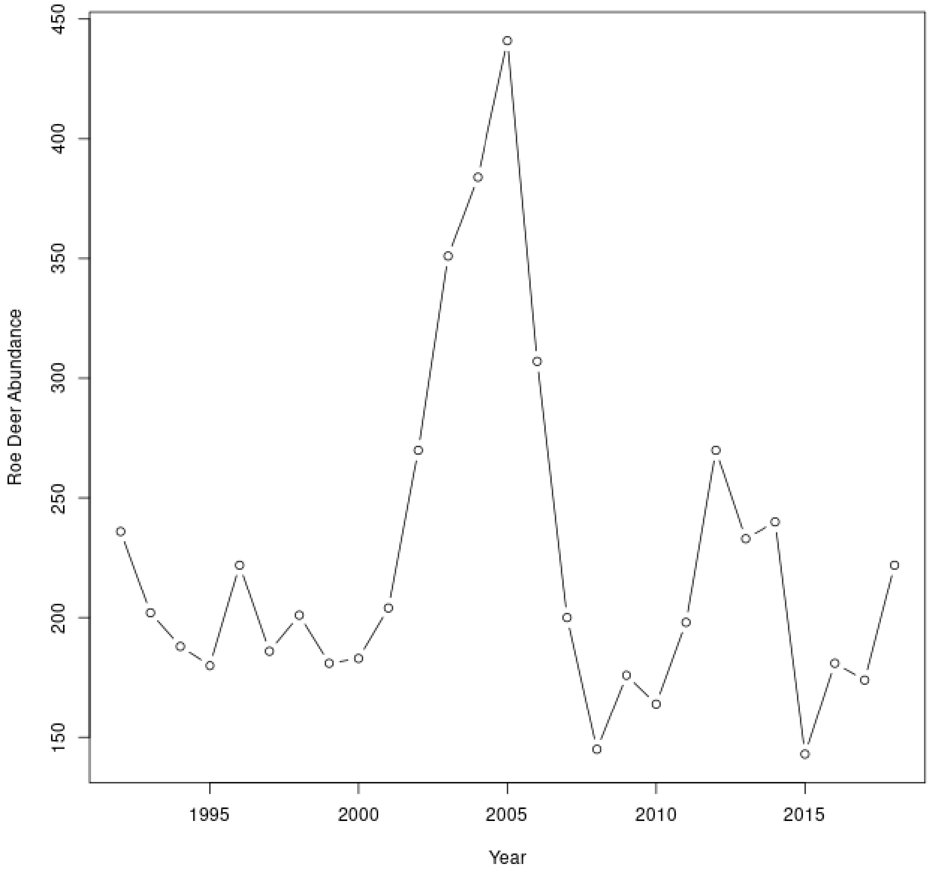

We used the same model code as in the previous section, but we replaced the additionnal variable with the density. Again, we fit the model with `nimble`. WARNING: THIS CALCULATION TAKES A VERY LONG TIME (more than one hour) !!!! Note that, as for the previous model, we also have included the results of this calculation as the dataset `resultsModelDensity` of the package, so that the reader does not need to launch this function to reproduce later calculations:

```
TicksDataP2 <- TicksData
TicksDataP2$VARIABLES <- fticks$density-mean(fticks$density)

set.seed(123)
resultsModelDensity <-
    nimbleMCMC(code = codePaperModelP1, constants = TicksConst,
               data = TicksDataP2, inits = linits,
               nchains = 4, niter = 51000, nburnin=1000, thin=20,
               summary = TRUE, samplesAsCodaMCMC=TRUE,
               monitors = c("delta_u","gammav", "sigma_u","gamman",
                            "intercept","phi","sigma_s","beta0p",
                            "beta1p","beta2p"))
```

The estimated parameter for the density is displayed below:

```
resultsModelDensity$summary$all.chains["gamman",]

##          Mean       Median      St.Dev.    95%CI_low
##   0.002176036  0.002184846  0.001681571 -0.001105672
##     95%CI_upp
##   0.005545895
```

Our data do not allow to demonstrate an effect of the density on the infestation rate.

3.3 Effect of habitat type

Finally, we assessed the effect of habitat on the instantaneous infestation rate, by adding random habitat effects in equation 3. We programmed the model below, and prepared the data for the fit. Note that only animals captured from 1992 to 2014 have habitat data reported (habitat is unknown for animals captured later).

```
codePaperModelHab <- nimbleCode({

    ## Priors
    intercept ~ dnorm(0,sd=20)
    gammah ~ dnorm(0,sd=20)
    gammav ~ dnorm(0,sd=20)
    beta0p ~ dnorm(0,sd=20)
    beta1p ~ dnorm(0,sd=20)
    beta2p ~ dnorm(0,sd=20)
    for (i in 1:3) {
        sigma_u[i]~dunif(0,100)
    }
    for (h in 1:5) {
        gammahab[h]~dnorm(0,sigma_hab)
    }
    sigma_s~dunif(0,100)
    sigma_hab~dunif(0,100)
    phi~dunif(0,100);


    ## Year random effects
    for (i in 1:nyears) {
        delta_u[i] ~ dnorm(intercept, sd=sigma_u[pery[i]]);
    }

    ## Because we work on centred data for body surface area
    ## (for better mixing)
    beta0 <- beta0p - beta1p*xbar + beta2p*pow(xbar,2.0)
    beta1 <- beta1p - 2*beta2p*xbar
    beta2 <- beta2p


    ## Integration of body surface area
    for (i in 1:nobs) {

        ## Expected bsa
        yhat[i] <- beta0p + beta1p*AGEc[i] + beta2p*pow(AGEc[i],2.0)
        SURFACE[i] ~ dnorm(yhat[i], sd=sigma_s)
        epsilon[i] <- SURFACE[i] - yhat[i]

        ## Integration
        Ej[i] <- step(5-AGE[i])*(beta0*AGE[i] + (beta1/2.0)*pow(AGE[i],2.0) +
                                 (beta2/3.0)*pow(AGE[i],3.0) +
                                 epsilon[i]*AGE[i]) +
            (1-step(5-AGE[i]))*((5.0*beta0 + 12.5*beta1 + (125.0/3.0)*beta2 + 5.0*epsilon[i]) +
                                exp(gammav)*(beta0*AGE[i] + (beta1/2.0)*pow(AGE[i],2.0) +
                                             (beta2/3.0)*pow(AGE[i],3.0) +
                                             epsilon[i]*AGE[i]) -
```

```
                                exp(gammav)*(5.0*beta0 + 12.5*beta1 +
                                             (125.0/3.0)*beta2 + 5.0*epsilon[i]))

        ## Expectation
        Lambdaj[i] <- Ej[i] * exp(delta_u[YEAR[i]]+gammah*HUMIDITY[i]+gammahab[HABITAT[i]])


        ## Likelihood
        TICK_CLASS[i]~dticks(Lambdaj[i], phi)
    }

})


fticksh <- fticks[!is.na(fticks$habitat),]

## Year as an integer variable
int_yearh <- as.integer(fticksh$year-(min(fticksh$year)-1))

## period for each year
ylh <- unique(fticksh[,c("year","lothar")])
peryh <- ylh$lothar[order(ylh$year)]


## Constants
TicksConsth <- list(nobs=nrow(fticksh), nyears=max(int_yearh), YEAR=int_yearh, pery=peryh,
                    HABITAT=as.integer(fticksh$habitat))

## Data
TicksDatah <- list(TICK_CLASS=as.integer(fticksh$ticks),
                   AGE=fticksh$age,
                   xbar=mean(fticksh$age),
                   AGEc=fticksh$age-mean(fticksh$age),
                   SURFACE=fticksh$bsa,
                   HUMIDITY=fticksh$humidity-mean(fticksh$humidity))

## Starting values
inits <- list(beta0p = 0.17, beta1p = 0.01, beta2p = -0.0007, gammahab=rep(0,5),
              gammav = -0.8, gammah=0, intercept = 2.8, phi = 0.79,
              sigma_u = c(0.64, 0.64, 0.64), sigma_s = 0.02,
              delta_u = c(2.07, 2.91, 2.79,
                          2.82, 2.57, 3.47, 2.85, 2.77, 2.59, 2.4, 1.45, 3.01, 2.5,
                          2.68, 2.76, 2.76, 2.71, 3.41, 2.43, 2.4, 2.59, 2.19, 2.63,
                          1.72, 2.12, 2.59, 2.19)[1:23])

## We use the same for the 4 chains
linits <- list(inits,inits,inits,inits)
```

Again, we fit the model with `nimble`. WARNING: THIS CALCULATION TAKES A VERY LONG TIME (more than one hour) !!!! Note that, as for the previous model, we also have included the results of this calculation as the dataset `resultsModelHabitat` of the package, so that the reader does not need to launch this function to reproduce later calculations:

```
set.seed(123)
resultsModelHabitat <-
    nimbleMCMC(code = codePaperModelHab, constants = TicksConsth,
               data = TicksDatah, inits = linits,
```

```
                nchains = 4, niter = 51000, nburnin=1000, thin=20,
                summary = TRUE, samplesAsCodaMCMC=TRUE,
                monitors = c("delta_u","gammav", "sigma_u","gammahab",
                             "intercept","phi","sigma_s","beta0p",
                             "beta1p","beta2p"))
```

We present the estimated habitat parameters below:

```
re <- resultsModelHabitat$summary$all.chains
round(re[grep("gammahab",rownames(re)),],2)

##              Mean Median St.Dev. 95%CI_low 95%CI_upp
## gammahab[1] -0.05  -0.05    0.11     -0.28      0.16
## gammahab[2]  0.06   0.06    0.11     -0.15      0.29
## gammahab[3] -0.09  -0.08    0.11     -0.31      0.12
## gammahab[4]  0.10   0.10    0.12     -0.12      0.36
## gammahab[5] -0.01  -0.01    0.11     -0.22      0.21
```

None of the parameters are significantly different from 0. Our data do not allow to demonstrate the effect of the habitat on the infestation rate.

References

[GEL92] Gelman, A. and Rubin, D. B. 1992. Inference from iterative simulation using multiple sequences. *Statistical Science*, 7, 457–472.

[VAL17] de Valpine, P., D. Turek, C.J. Paciorek, C. Anderson-Bergman, D. Temple Lang, and R. Bodik. 2017. Programming with models: writing statistical algorithms for general model structures with NIMBLE. *Journal of Computational and Graphical Statistics* 26: 403-413.

[VAL21] de Valpine P, Paciorek C, Turek D, Michaud N, Anderson-Bergman C, Obermeyer F, Wehrhahn Cortes C, Rodriguez A, Temple Lang D, Paganin S (2021). *NIMBLE User Manual*. doi: 10.5281/zenodo.1211190 (URL: https://doi.org/10.5281/zenodo.1211190), R package manual version 0.11.1, https://r-nimble.org.

[VEN02] Venables, W. N., and Ripley, B. D. 2002 Modern applied statistics with S-Plus. Fourth Edition. Springer: Berlin.
